# Macrophages in the Inflammatory Phase following Myocardial Infarction: Role of Exogenous Ubiquitin

**DOI:** 10.3390/biology12091258

**Published:** 2023-09-20

**Authors:** Paige L. Shook, Mahipal Singh, Krishna Singh

**Affiliations:** 1 Department of Biomedical Sciences, James H. Quillen College of Medicine, East Tennessee State University, Johnson City, TN 37614, USA; shookpl@etsu.edu (P.L.S.); singhm@etsu.edu (M.S.); 2 Center of Excellence in Inflammation, Infectious Disease and Immunity, James H. Quillen College of Medicine, East Tennessee State University, Johnson City, TN 37614, USA; 3 James H. Quillen Veterans Affairs Medical Center, Mountain Home, TN 37684, USA

**Keywords:** heart, myocardial infarction, inflammation, macrophage, ubiquitin, polarization, migration, efferocytosis

## Abstract

**Simple Summary:**

Heart disease is a major cause of death worldwide. A common implication of heart disease is myocardial infarction, commonly known as heart attack. Generally, myocardial infarction happens when the blood supply to a portion of the heart is reduced or blocked due to fat buildup. This blockage of blood flow damages part of the heart muscle and decreases heart function. To compensate for this injury, the heart initiates remodeling which consists of three overlapping phases of inflammation, proliferation, and maturation. This review focusses on the inflammatory phase, specifically the roles of macrophages and ubiquitin in heart repair following myocardial infarction, and discusses how ubiquitin could impact macrophage function. Macrophages are specialized blood cells with a critical role in wound healing. Ubiquitin is a highly evolutionarily conserved protein found in plasma. Plasma ubiquitin levels are increased in several disease states, including heart disease.

**Abstract:**

Cardiovascular disease (CVD) is one of the leading causes of death worldwide. One of the most common implications of CVD is myocardial infarction (MI). Following MI, the repair of the infarcted heart occurs through three distinct, yet overlapping phases of inflammation, proliferation, and maturation. Macrophages are essential to the resolution of the inflammatory phase due to their role in phagocytosis and efferocytosis. However, excessive and long-term macrophage accumulation at the area of injury and dysregulated function can induce adverse cardiac remodeling post-MI. Ubiquitin (UB) is a highly evolutionarily conserved small protein and is a normal constituent of plasma. Levels of UB are increased in the plasma during a variety of pathological conditions, including ischemic heart disease. Treatment of mice with UB associates with decreased inflammatory response and improved heart function following ischemia/reperfusion injury. This review summarizes the role of macrophages in the infarct healing process of the heart post-MI, and discusses the role of exogenous UB in myocardial remodeling post-MI and in the modulation of macrophage phenotype and function.

## 1. Introduction

Cardiovascular disease (CVD) is one of the leading causes of morbidity and mortality worldwide [1]. One of the most common implications of CVD is myocardial infarction (MI) [1]. MI is an ischemic injury in the myocardium, leading to cell death and adverse cardiac remodeling. There are multiple phases of cardiac remodeling, including the inflammatory, proliferative, and maturation phases [2,3]. The inflammatory response following MI is essential for the transition into the proliferative and maturation phases of cardiac repair, yet unresolved inflammation is a major mediator of advanced heart failure [4]. Proper regulation of the immune response holds promise to improve patients’ outlook. Macrophages are involved in the inflammatory response and are essential modulators of wound healing and repair, providing pro-inflammatory signaling at the onset of injury, clearing the wound from cellular debris, and then promoting anti-inflammatory signaling at later stages, aiding in scar formation through processes such as fibroblast activation and angiogenesis [5,6].

Ubiquitin (UB) is a highly evolutionarily conserved small protein with a molecular weight of ~8.5 kDa. It is found in all eukaryotic cells. The major function of intracellular UB is to mark the proteins for degradation via the ubiquitin–proteasome system [7,8]. Ubiquitination of intracellular proteins can also affect their cellular localization and function [9,10]. Normal plasma, urine, and cerebrospinal fluids are shown to have small amounts of UB. Normal human serum has UB concentrations ranging from ~30 to 120 ng/mL (depending on storage conditions), while mouse serum has a UB concentration of ~16 ng/mL [7,11,12]. Increased plasma levels of UB are demonstrated in a variety of pathological conditions, including ischemic heart disease [7,13]. Several in vitro and in vivo studies provide evidence that extracellular/exogenous UB (eUB) has pleiotropic functions, including modulation of cell survival, tissue injury, and immune response [11,14,15,16,17,18,19]. In the heart, treatment of mice with eUB was associated with decreased inflammatory response 3 days following ischemia reperfusion (I/R) [17]. Other studies have provided evidence that eUB has the potential to affect macrophage phenotype and function [20,21]. Therefore, the main objective of this review article is to summarize the function of macrophages (signaling, migration, phagocytosis, and efferocytosis) in the heart post-MI, and then discuss the role of eUB in modulation of cardiac remodeling and macrophage phenotype and function.

## 2. Myocardial Infarction and Remodeling

An MI is an ischemic injury to the heart. Ischemia results in myocardial cell death and leads to cardiac remodeling, which occurs in distinct but overlapping phases of inflammation, proliferation, and maturation [2,3]. The initial onset of ischemia occurs via an occlusion of a coronary artery which prevents blood flow to the myocardial tissue. This induces cardiac cell death due to necrosis or apoptosis. The terminally differentiated nature of myocytes entails loss of myocardial tissue post-MI [22]. To compensate for this loss and restore function, the heart undergoes remodeling. In response to cell death, the phase of inflammation begins and signals for infiltrating cells such as neutrophils and macrophages to clear the infarcted area of cellular debris [23]. The inflammatory phase can become exaggerated and initiate adverse effects on remodeling, potentially increasing the infarct size [2,23]. The proliferative phase of remodeling occurs after the onset of inflammation and leads to the development of scar tissue. During this phase, fibroblasts migrate to the area of infarct, transform into myofibroblasts, and deposit collagen type III to form the fibrotic scar [24,25]. This process can also become maladaptive, leading to excessive and/or disorganized deposition of extracellular matrix (ECM) with reduced cardiac contractile ability [26,27]. Contractile dysfunction can increase hemodynamic stress on ventricle walls, leading to hypertrophic remodeling. Myocardial hypertrophy occurs throughout to compensate for increased hemodynamic stress, although hypertrophy itself can become detrimental [28]. Finally, the maturation phase includes the continual remodeling of the scar tissue and replacing collagen III with collagen I, and the cross linking of collagen fibers to increase tensile scar strength [24,25]. Cardiac remodeling is essential to the repair of the heart post-MI. However, a maladaptive response through persistent inflammation, excess apoptosis, fibrosis, and cardiac hypertrophy can increase the risk of successive injury, heart failure, and death [2,22,28].

## 3. Inflammation in the Heart

The inflammatory response post-MI is essential to transition into cardiac repair. The duration, size, and regulation of the inflammatory phase can be indicative of an effective or detrimental immune response. Unresolved inflammation can further drive infarct expansion and adverse cardiac remodeling [2]. In the heart, several signaling pathways initiate inflammation signaling, including reactive oxygen species (ROS), complement cascade, and damage-associated molecular patterns (DAMPs) [29]. Resident cardiac cells and circulating immune cells then amplify the inflammatory response through the release of pro-inflammatory cytokines and chemokines such as interleukin-1α (IL-1α), IL-6, chemokine ligand 5 (CCL5), and monocyte chemoattractant protein-1 (MCP-1) [29,30]. Neutrophils are the first cells to mobilize to the area of injury and use methods of degranulation, phagocytosis, and neutrophil extracellular traps (NET) to clear the infarcted area. Neutrophils also produce ROS which can increase cell death, most notably at the infarct border zone [31,32]. Therefore, increased infiltration, activation, and slower clearance of neutrophils results in poorer prognosis [31,33]. Macrophages also infiltrate the infarcted myocardium. In the infarcted cardiac tissue, macrophages produce pro-inflammatory proteins, phagocytose apoptotic cells and debris, produce factors to stimulate angiogenesis and fibrosis, and present antigens for adaptive immunity [6]. Macrophages are essential to the resolution of the inflammatory phase, but excessive macrophage accumulation at the area of injury can increase deleterious inflammation [34]. T cells, a part of the adaptive immune response post-MI, can also regulate the innate immune response via macrophage interactions. In the heart post-MI, CD8^+^ T cells have both beneficial and detrimental effects through the signaling relationship with macrophages. In CD8^+^ T-cell-deficient mice there is reduced clearance of apoptotic and necrotic cellular debris and associates with increased soluble tyrosine-protein kinase MER, which can inhibit macrophage phagocytosis [35]. CD4^+^ regulatory T cells may play a role in macrophage differentiation into an anti-inflammatory (pro-healing) phenotype, as depletion of CD4^+^ regulatory T cells results in preferential pro-inflammatory polarization of myocardial macrophages [36]. CD8^+^ T cells can also encourage overactivation of macrophages, leading to increased time of myocardial recovery [35]. Overall, persistent, and damaging inflammation post-MI could place patients at risk for dysfunctional cardiac repair.

## 4. Macrophages and the Heart

Macrophages are immune cells that work in the innate and adaptive immune system. The roles of macrophages include inflammatory and anti-inflammatory signaling, phagocytosis, production of proangiogenic and pro-reparative factors, and antigen presentation to lymphocytes for adaptive immunity [6]. In the heart, resident macrophages originate from the embryonic yolk sac, locally proliferate, and are involved in routine tissue maintenance [6,37]. There are several macrophage markers used to distinguish macrophage subsets in the heart at steady-state and post-MI. These markers include TIMD4 (T-cell immunoglobulin and mucin domain containing 4), MHC-II (major histocompatibility complex II), LYVE1 (lymphatic vessel endothelial hyaluronan receptor 1), CCR2 (C-C motif chemokine receptor 2), Ly6C (lymphocyte antigen 6), or CX3CR1 (C-X3-C Motif Chemokine Receptor 1) [6,38,39]. TIMD4^+^ expression is indicative of a self-renewing population of resident macrophages, while CCR2^+^ expression indicates recruited macrophages renewed by circulating monocytes [38]. The primary role of resident cardiac tissue macrophages is routine tissue maintenance [6]. However, post-MI, the self-renewing TIMD4^+^ macrophages limit adverse myocardial remodeling potentially through their distinct reparative gene expression (genes such as: TIMD4, LYVE1, IGF1 and FOLR2). Depletion of resident macrophages impairs cardiac function and exacerbates infarct healing [38]. In pressure overload-induced cardiac remodeling, resident macrophages are shown to stimulate angiogenesis and limit cardiac fibrosis [40]. A subpopulation of CCR2^+^ tissue resident macrophages derived from circulating monocytes are suggested to be involved in inflammation and play a role in the recruitment of monocyte-derived macrophages to the heart post-I/R [41]. The TLR9/MyD88-mediated production of chemokines CXCL2 (CXC chemokine ligand 2) and CXCL5 from CCR2^+^ tissue resident monocyte-derived macrophages are important for extravasation of neutrophils into cardiac tissue [42]. These subsets of resident macrophages are essential to myocardial repair post-MI. However, immediately following MI, the resident macrophage population is significantly reduced, predominantly due to local cell death. Macrophages derived from blood monocytes replenish the macrophage pool [37]. The loss of resident macrophages is not compensated for, as they are pro-resolving and have distinct gene expressions with specialized functions that are not mirrored in the recruited macrophage population [6,38]. In response to myocardial injury, circulating peripheral blood monocytes are recruited from the bone marrow and the spleen, migrate to the heart via CCL2/CCR2 signaling, and differentiate into macrophages [6,43]. These infiltrating macrophages display various phenotypes depending on environmental stimuli and are important mediators of cardiac remodeling involved in inflammatory as well as the reparative signaling [5]. This regulation is achieved through the release of pro- and anti-inflammatory mediators by polarized macrophages, as well as via functions such as migration and phagocytosis.

## 5. Macrophage Phenotype and Function

### 5.1. Macrophage Polarization

Macrophage polarization post-MI determines the functional roles of these immune cells. Traditional terminology refers to pro-inflammatory (M1) and anti-inflammatory (M2) phenotypes. However, the extent of macrophage polarization is not entirely encompassed within these two categories. The expression of pro-inflammatory and anti-inflammatory genes varies throughout cardiac remodeling post-MI. The polarization of macrophages post-MI has been mapped over a time continuum by Mouton et al. 2018 [5]. Macrophages have largely proinflammatory gene expression 1 day post MI. They express genes related to a phagocytic, proliferative, and metabolic reprogramming profile 3 days post-MI, then they have reparative markers with highly expressed genes associated with scar formation 7 days post-MI [5].

**Figure 1:**
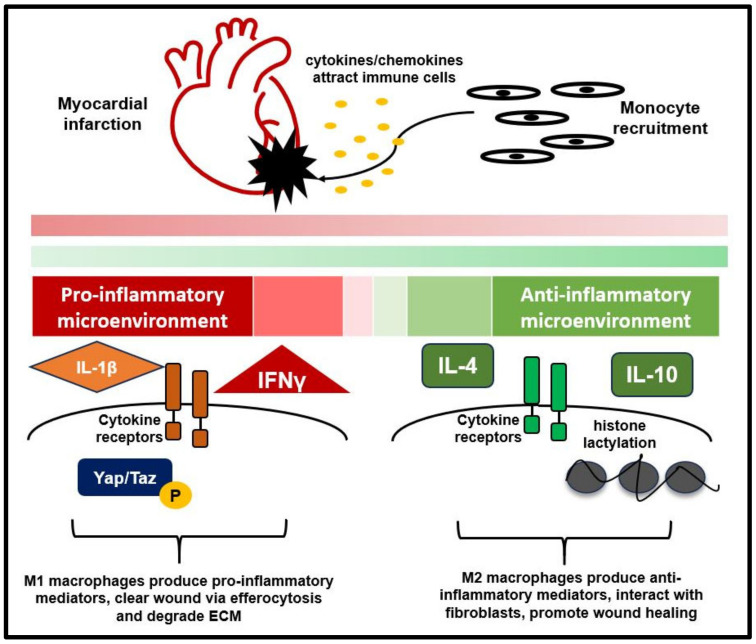
Macrophage polarization post-MI. Following MI, chemokines/cytokines released from cardiac resident and circulating immune cells are major mediators of immune cell recruitment. Monocytes stored in the bone marrow or spleen are released into circulation and migrate to the heart. At the area of injury, monocytes differentiate to macrophages and can be polarized into a range of phenotypes. M1 macrophages produce pro-inflammatory mediators, work in the initial wound clearance of apoptotic cells via efferocytosis and degrade ECM. M2 macrophages produce anti-inflammatory mediators, interact with fibroblasts, and promote wound healing.

Early following MI (1 day), the pro-inflammatory phenotype of macrophages predominates. In this acute inflammatory phase, the primary function of macrophages is to engulf cellular debris, degrade ECM, and release inflammatory cytokines (Figure 1) [44,45]. IL-1β and interferon-γ (IFNγ) are the primary macrophage-derived mediators of this inflammatory environment [5]. IL-1β is a pro-inflammatory cytokine released by immune cells after their exposure to pathogen-associated molecular patterns (PAMPs) or DAMPs [46]. IL-1β binding to the type 1 IL-1 receptor activates nuclear factor kappa-light-chain-enhancer of activated B cells (NF-κB), C-Jun N-terminal kinase (JNK), and p38 mitogen-activated protein kinase (MAPK) [47]. Activation of these signaling molecules induces expression of genes such as IL-6, IL-8, MCP-1 (monocyte chemoattractant protein -1), COX-2 (cyclooxygenase 2), IκBα (nuclear factor of kappa light polypeptide gene enhancer in B-cells inhibitor, alpha), IL-1α, IL-1β, and MKP-1 (mitogen-activated protein kinase phosphatase-1) [47]. IFNγ signals through the IFNγ receptor activating Janus kinase (JAK) and signal transducer and activator of transcription 1 (STAT1). Upon phosphorylation, STAT1 is translocated to the nucleus and activates the transcription of interferon-stimulated genes (ISG) [48]. ISGs can encode chemokines, antigen-presenting molecules such as MHCs, and phagocytic receptors [48]. In the acute inflammatory phase (1-day post-MI), macrophages have a distinctive expression of genes associated with IL-1, TNF, NF-κB, MAPK (mitogen-activated protein kinase), STAT5, and suppressor of cytokine signaling 2 (SOCS2) signaling pathways [5].

As the inflammatory phase progresses, macrophage phenotype continues to shift. By 3 days post MI, macrophages display changes in mitochondrial function, increased phagocytosis, and phagosome maturation compared to residential macrophages. Additionally, these macrophages start to express proliferative markers [5]. By 7 days post-MI, the reparative M2 macrophage phenotype dominates, with expression of genes related to ECM remodeling and scar formation (Figure 1) [5]. Transition towards M2 polarization can be a result of the tissue microenvironment. Some of the contributing factors include glucocorticoids, prostaglandin derivatives, adenosine, anti-inflammatory cytokines, and growth factors [49]. The phagocytosis of apoptotic cells by macrophages is also suggested to play a role in the transition towards the M2 phenotype [50]. The reparative phenotype of macrophages associates with the production of several factors linked with cell proliferation and angiogenesis, including platelet-derived growth factor (PDGF), TGF-β1, insulin-like growth factor 1 (IGF-1), and vascular endothelial growth factor-α (VEGF-α). In addition, macrophages interact with fibroblasts to promote wound healing and closure (Figure 1) [51]. Therefore, the function of macrophages late post-MI can promote repair via the expression of factors for cellular proliferation and angiogenesis, and interactions with fibroblasts to modulate ECM and the formation of scar tissue.

Macrophage polarization is a potential target to promote myocardial repair post-MI. A timely shift to the pro-resolving phenotype of macrophages may reduce excess inflammation and hasten infarct healing. Several pathways have been suggested to influence pro-resolving macrophage phenotype post-MI. Histone lactylation promotes pro-reparative gene expression in monocyte-derived macrophages, and improves cardiac function post-MI [52]. Osteopontin (OPN) produced by pro-resolving macrophages through the activation of the IL-10-STAT3-galectin-3 axis has been shown to increase phagocytic clearance of apoptotic cells and promote reparative fibrosis following MI [53]. Deletion of CD226, a member of the immunoglobulin superfamily, in macrophages favors M2 polarization. CD226 knock out mice exhibit improved wound healing and cardiac function post-MI [54]. Yes activated protein (YAP) and transcription coactivator with PDZ-binding motif (TAZ) are involved in the pro-inflammatory activation of macrophages. Genetic deletion of YAP/TAZ in macrophages leads to reduced fibrosis, hypertrophy, increased angiogenesis, and improved cardiac function post-MI [55]. Treatment with IL-10, an anti-inflammatory cytokine, has been shown to promote expression of M2 markers (Arg1, Ym1, and TGFβ), reduce inflammation, and improve cardiac function post-MI [56]. Macrophage-specific knock-out of Lgr4, a leucine-rich repeat-containing G protein-coupled receptor, in a mouse model of MI resulted in infarct macrophages with less pro-inflammatory gene expression, attenuated inflammation, and improved myocardial healing [57]. Thus, multiple pathways govern the polarization of macrophages post-MI. Polarization towards a pro-reparative (M2) phenotype may be a useful therapeutic target to improve myocardial repair.

### 5.2. Macrophage Migration

**Figure 2:**
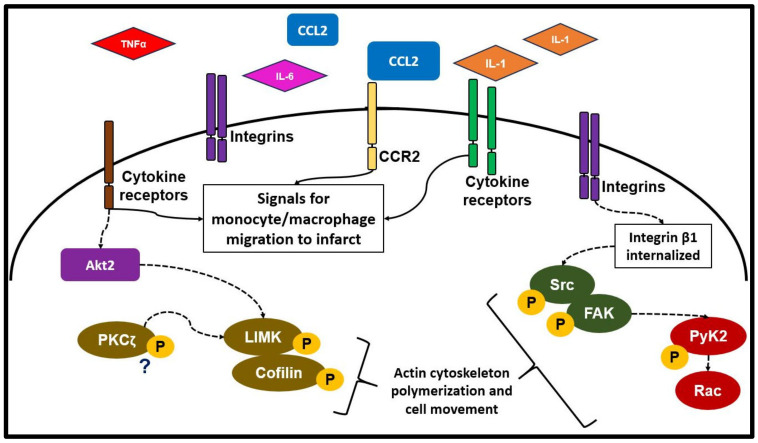
Macrophage migration post-MI. Following MI, expression of chemokines/cytokines such as IL-1, IL-6, TNF-α, and CCL2 initiates migration of immune cells to the area of infarct. CCL2, one of the major chemokine mediators in monocyte migration to the heart post-MI, signals through the CCR2 receptor. Integrins are essential to the migration process. The internalization of integrin β1 can activate FAK-Src/PyK2-Rac, leading to alterations in actin polymerization and the resultant cell movement. Akt2 activation may affect actin polymerization through the phosphorylation of PKCζ and LIMK/cofilin, thereby affecting chemotaxis. Migration is essential for immune cell recruitment to the heart post-MI.

The migration of macrophages to the site of injury is essential for wound repair post-MI. After the initiation of the inflammatory phase, cytokines (such as TNF-α, IL-1, IL-6) released from cardiac resident and circulating immune cells are suggested to be involved in further recruitment of immune cells [30]. The complement system may also play a role in macrophage migration through component5a (C5a) signaling [58,59]. One of the most well studied mechanisms of monocyte–macrophage migration to the infarcted myocardium involves monocyte recruitment to the area of tissue injury via MCP-1/CCL2 signaling through CCR-2 (Figure 2) [60]. Then, adhesion to the ECM can initiate the expression of cytokines including TNFα, macrophage-colony stimulating factor (M-CSF), PDGF, IL-1, IGF, TGFα, and TGFβ, which promotes the conversion from monocytes to macrophages [61].

During migration, macrophages will encounter both 2D and 3D environments. Two-dimensional migration includes migration along the inner vessel walls and basement membranes. Three-dimensional migration occurs within interstitial matrices [62]. Macrophages can use amoeboid or mesenchymal migration to travel through these environments. Amoeboid migration constitutes the movement of rounded cells along the ECM. Mesenchymal migration occurs with elongated cells that travel through and degrade ECM [62]. Integrins found on the surface of macrophages are essential for adhesion to the ECM and are significantly involved in 2D migration [63]. The differential expression of adhesion integrins α_M_β_2_ and α_D_β_2_ has been shown to impact macrophage migration and retention using a 3D migration assay [64]. Additional signaling mechanisms that promote macrophage migration may include internalization of integrin β1 and activation of FAK/Src and Pyk2-Rac signaling pathways [65]. Activation of these signaling pathways is suggested to be involved in macrophage polarization and migration [65]. In addition, activation of Akt2, a serine–threonine kinase, is shown to be involved in macrophage chemotaxis [66]. siRNA-mediated downregulation of Akt2 expression in THP-1 monocytes and mouse peritoneal macrophages inhibited CSF-1-induced phosphorylation of PKCζ and LIMK/Cofilin, leading to defective actin polymerization and reduced chemotaxis [66] (Figure 2).

The migration of macrophages to the ischemic myocardium is essential to cardiac repair. This is evidenced by the observation that depletion of macrophages in a murine cryoinjury model to induce left ventricular (LV) damage results in reduced wound healing, increased adverse cardiac remodeling, and enhanced mortality [67]. Conversely, the injection of activated macrophages into a rat model of infarcted myocardium improves cardiac remodeling and function post-MI [68]. Therefore, macrophages are key mediators in cardiac remodeling. However, the excessive infiltration of macrophages as seen in a model of cardiomyocyte overexpression of G protein-coupled receptor kinase 5 (GRK5) can lead to chronic inflammation, increased adverse LV remodeling, and impaired LV function post-MI [69]. Hydrogen sulfide, a gasotransmitter, promotes the early, not late, infiltration of macrophages to the heart post-MI by enhancing their migratory potential. This time-dependent macrophage recruitment may be one potential mechanism whereby treatment with hydrogen sulfide significantly improves cardiac remodeling and function post-MI [65]. Therefore, a timely infiltration of macrophages may be beneficial in wound repair and improved cardiac outcome late post-MI. Of note, hydrogen sulfide can be a “double-edged sword” with beneficial effects at lower concentrations, but potentially harmful effects at higher concentrations, due to its pro-oxidant and cytotoxic responses [70].

### 5.3. Macrophage Phagocytosis/Efferocytosis

Clearance of apoptotic cells and cellular debris by macrophages is essential to wound healing. Decreased phagocytic activity can exacerbate cardiac injury and lead to secondary necrosis of cells. Necrosis causes the release of the cells’ cytotoxic contents, leading to further elevation of the immune response and local cell death [71]. Post-MI, defective macrophage phagocytosis has been linked to increased adverse myocardial remodeling, decreased cardiac function, and enhanced mortality rates [39,67,72]. Therefore, proper clearance of apoptotic cells, termed efferocytosis, by macrophages is essential to the effective regulation of the inflammatory phase and improved cardiac remodeling.

**Figure 3:**
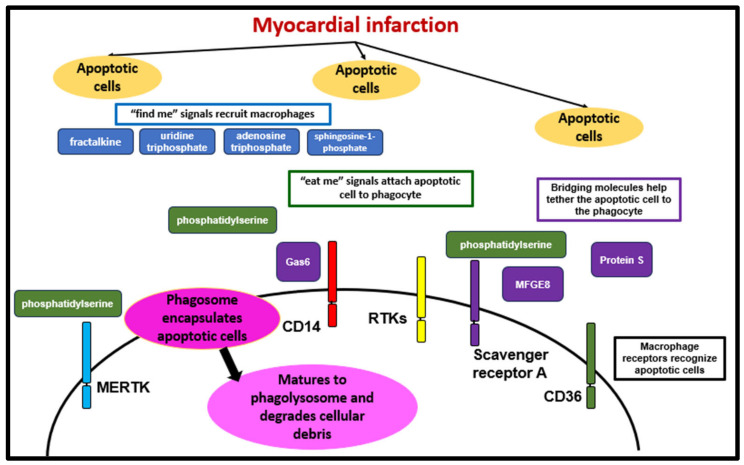
Macrophage efferocytosis post-MI. Following MI, macrophages are the key cell-type to remove apoptotic cells through a process termed efferocytosis. Apoptotic cells recruit macrophages via “find me” signals, such as fractalkine, uridine triphosphate, adenosine triphosphate, and sphingosine-1-phosphate. Then, macrophages use “eat me” signals, like phosphatidylserine, to attach to the apoptotic cell. Bridging molecules including protein S, Gas6, and milk fat globule-EGF factor 8 (MFGE8) further aid in tethering the apoptotic cell to the macrophage. Macrophage receptors (such as myeloid epithelial-reproductive tyrosine kinase (MERTK), CD14, receptor tyrosine kinases (RTKs), scavenger receptor A and scavenger receptor B (CD36)) for efferocytosis recognize apoptotic cells. After recruitment, recognition, and tethering, macrophages engulf apoptotic cells into phagosomes, which mature into phagolysosomes to digest cellular debris.

Specific signals under the terminology of “find me”, “eat me”, and “don’t eat me” allow for the recognition and uptake of apoptotic cells and prevent the efferocytosis of healthy cells [73]. To ensure the proper clearance of only the intended targets, efferocytosis mechanisms include recruitment, recognition and tethering, engulfment, and degradation [73]. Apoptotic cells use “find me” signals to recruit macrophages [73]. The “find me” signals emitted by apoptotic cells include nucleotides such as adenosine triphosphate and uridine triphosphate. Other “find me” signals include lysophosphatidylcholine, sphingosine-1-phosphate, and fractalkine (CX3CL1) [73,74]. Then, “eat me” signals near the phagocyte indicate that the cell is apoptotic, leading to macrophage cytoskeletal rearrangement and engulfment. The most potent “eat me” signal generally found on apoptotic cells is externalized phosphatidylserine [73,74]. Macrophage receptors responsible for recognizing apoptotic cells include the scavenger receptor A, scavenger receptor B (also known as CD36), vitronectin receptor, CD14 [75,76], brain-specific angiogenesis inhibitor 1 (BAI1) [74], T-cell membrane protein (Tim) family of receptors including TIM1 and TIM4 [75], as well as the receptor tyrosine kinases (RTKs) found on the cell surface, including TAM proteins TYRO3, AXL, and myeloid epithelial-reproductive (MER) tyrosine kinase (encoded by MERTK) (Figure 3) [74]. Bridging molecules then aid in tethering the apoptotic cell to the phagocyte. Milk fat globule-EGF factor 8 (MFGE8) is a bridging molecule with roles in cytoskeletal rearrangement and production of anti-inflammatory cytokines. Other molecules include annexin 1, growth arrest specific gene 6 (Gas6), and protein S. Then, the “don’t eat me” ligands include CD47, and CD300a [73,74]. Decreased expression of CD47 and CD300a on the surface of apoptotic cells can indicate the need for clearance [73,77]. After the appropriate recruitment, recognition, and tethering, there is engulfment and degradation of apoptotic cells. A phagosome forms as a vesicle enclosing the ingested particles, then the phagosome matures [76]. In the maturation process, there are early and late stages of phagosome–endosome formation followed by the development of the phagolysosome, which is responsible for the degradation of the engulfed particles (Figure 3) [76,78]. After the phagocytosis of apoptotic cells, macrophages produce anti-inflammatory mediators, such as TGF-β, prostaglandin E2 (PGE2), platelet activating factor (PAF), and IL-10; and other specialized pro-resolving mediators such as long-chain fatty acid-derived lipids including lipoxin A4, and resolvins D1, D2, and E2. Additionally, the expression of pro-inflammatory factors such as inducible nitric oxide synthase (iNOS), TNF-α, IL-1, IL-8, and IL-12 is suppressed [71,73].

In the heart, efferocytosis plays an important role in cardiac repair post-MI. In a murine cryoinjury model, macrophage-depleted hearts exhibited the presence of non-resorbed cellular debris 4 weeks after injury, and macrophage depletion associated with higher mortality and increased LV dilation and wall thinning [67]. The efferocytosis of apoptotic neutrophils by macrophages is an essential function during acute inflammation post-MI. Treatment with exogenous IL-4 24 h post-MI significantly increases anti-inflammatory macrophage gene expression, and enhances ex vivo efferocytosis of neutrophils, with an accordant increase in gene expression related to macrophage phagocytosis including MERTK, Mrc1, and Fcgr2b expression [79]. Macrophage efferocytosis is also suggested to play a role in the production of vascular endothelial growth factor C (VEGF-C), which has been shown to be involved in the suppression of macrophage pro-inflammatory activation and initiation of cardiac lymphangiogenesis [80]. Impairment of efferocytosis induced by a deficiency in lysosomal enzyme legumain (Lgmn), results in decreased efficiency to clear and degrade apoptotic cardiomyocytes, and exacerbation of cardiac function post-MI [39]. Macrophages lacking Smad3 exhibit reduced expression of MFGE8 and reduced phagocytosis. A myeloid cell-type-specific Smad3 knockout in mice resulted in increased adverse cardiac remodeling and mortality post-MI [81]. Additionally, MERTK is shown to be essential to the efferocytosis of apoptotic cardiac myocytes as deficiency of MERTK in macrophages suppresses efferocytosis, delays the resolution of inflammation, reduces systolic function, and increases infarct size post-MI [72]. Taken together, these studies suggest that multiple signaling molecules regulate the processing and degradation of apoptotic cells post-MI. Proper clearance of apoptotic cells by macrophages plays an important role in myocardial repair post-MI.

## 6. Ubiquitin and the Heart

### 6.1. Ubiquitin and Its Intracellular Functions

Ubiquitin (UB) is a ~8.5 kDa highly conserved protein. It is found in all eukaryotic cells [7]. The primary function of intracellular UB is to serve as a post-translational protein modifier [8]. The lysine residues in UB are used to form polyubiquitin chains on proteins to mark them for degradation via the UB-proteasome system (UPS). In a concerted action, E1 (UB-activating enzyme), E2 (UB-carrier or conjugating proteins), and E3 (UB-protein ligase) link UB chains onto cellular proteins [8]. However, UB can also be linked to cellular proteins as a monomer. This monoubiquitination of proteins can serve as a regulator for subcellular localization, protein–protein interactions, gene expression, and endocytosis of cell surface proteins, thereby affecting cellular homeostasis and survival [10].

### 6.2. Extracellular/Exogenous UB (eUB)

UB is a normal constituent of plasma, urine, and cerebrospinal fluid. A variety of pathological conditions increase serum UB levels [7]. Secretion in extracellular space by an exocytic mechanism from normal cells, necrosis of damaged cells, and hemolysis have been suggested as potential mechanisms for the presence of UB in bodily fluids [7,11,82]. eUB is suggested to have pleiotropic functions affecting immune modulation, cell survival, proliferation, chemotaxis, phenotype, and function of various cell types and tissues [7]. The G protein coupled receptor CXC chemokine receptor 4, CXCR4, is identified as a receptor for eUB [83]. Using AMD3100 (CXCR4 antagonist) and SDF-1α (a cognate ligand for CXCR4), Saini et al. demonstrated that eUB undergoes agonist-mediated endocytosis via CXCR4 [83].

### 6.3. Exogenous UB and the Heart

In a search to identify survival factor/s, an increased presence of UB was observed in the conditioned media of adult rat ventricular myocytes (ARVMs) treated with a β-adrenergic receptor (β-AR) agonist (isoproterenol, an apoptotic agent). Treatment of ARVMs with UB inhibited β-AR-stimulated activation of GSK-3β and JNK and increased apoptosis [84]. Use of biotin-labelled UB provided evidence for the uptake of eUB into the cells, while use of methylated UB (unable to form polyubiquitin chains) provided evidence that the protective effects of eUB are mediated via the monoubiquitination of cellular proteins [84]. UB treatment also inhibited hypoxia/reoxygenation (in vitro model to simulate myocardial I/R injury)-induced apoptosis in ARVMs. ARVMs express CXCR4. siRNA-mediated knockdown and CXCR4 antagonism using AMD3100 negated the anti-apoptotic effects of UB treatment in response to hypoxia/reoxygenation [18]. In H9C2 cardiac myoblast cell line, UB treatment also inhibited hypoxia-induced apoptosis. CXCR4 antagonism using AMD3100 inhibited the anti-apoptotic effects of eUB [85]. UB treatment is also shown to affect the phenotype and function of cardiac fibroblasts and microvascular endothelial cells. In cardiac fibroblasts, UB significantly reduced migration and proliferation, and enhanced the contraction of fibroblast-populated collagen pads while increasing the expression of α smooth muscle actin, a marker of myofibroblasts. The majority of these effects of eUB were inhibited by pretreatment with AMD3100 [86]. In isolated cardiac microvascular endothelial cells, UB treatment induced actin reorganization, enhanced migration into the wound, and increased tubular network formation and micro-vessel sprouting. Use of methylated UB enhanced tube network formation, suggesting a role for monoubiquitination [87]. Together, these in vitro studies suggest that eUB has the potential to affect cardiac cell survival, phenotype, and function via the involvement of the UB/CXCR4 axis. The effects of eUB are likely mediated via the monoubiquitination of cellular proteins.

Plasma levels of UB are shown to increase in patients with coronary heart disease. In patients with coronary heart disease, plasma UB levels correlated positively with levels of cardiac troponin I, C-reactive protein, and creatine kinase-MB. Additionally, plasma levels of UB were significantly higher in patients with acute MI versus stable angina pectoris and unstable angina pectoris [13], suggesting that change in plasma UB levels may reflect the progression of coronary heart disease. Using animal models of myocardial remodeling, UB treatment is shown to have cardioprotective effects with respect to heart function and remodeling parameters. Sympathetic nerve activity increases in the heart during hemodynamic stress and heart failure [88,89,90]. This exposes cardiac cells to norepinephrine, a primary neurotransmitter, and stimulation of β-AR. β-AR stimulation induces cardiac myocyte apoptosis in vitro and in vivo [91,92]. In addition, prolonged stimulation of β-AR axis creates infarct-like lesions, and induces hypertrophy and fibrosis in the heart [93,94]. In β-AR-stimulated mouse model of myocardial remodeling, UB treatment was associated with preservation of heart function and decreased cardiac myocyte apoptosis and myocardial fibrosis [95]. In a mouse model of myocardial I/R, UB treatment decreased inflammatory response and preserved heart function 3 days post-I/R [17]. Likewise, ex vivo UB treatment decreased infarct size and improved the functional recovery of the heart following global I/R [18]. Long-term beneficial effects of eUB are demonstrated by the observation of improved heart function and decreased myocardial fibrosis, apoptosis, and hypertrophy in UB-treated hearts 28 days post-I/R in mice [96]. Thus, UB treatment significantly improves cardiac function and reduces adverse cardiac remodeling parameters in mouse models of chronic β-AR stimulation and I/R injury.

## 7. Exogenous UB and Immune Modulation

### 7.1. Exogenous UB and the Immune Response

eUB has been suggested to have immunomodulatory effects. It has been shown to reduce inflammation and tissue injury in multiple models including endotoxemia, blunt polytrauma with lung injury, severe trauma, controlled cortical impact injury, lung I/R injury, and myocardial I/R injury [15,16,17,96,97,98,99]. In a swine endotoxemia model, UB treatment reduced mortality and prevented the development of pulmonary failure. UB treatment also inhibited LPS-induced TNF-α production in whole blood ex vivo [98]. In blunt polytrauma swine model consisting of bilateral femur fractures plus blunt chest trauma, UB treatment preserved arterial oxygenation, improved resuscitation, and reduced fluid shifts into tissues, including lung, heart, spleen, and jejunum. In addition, UB treatment reduced IL-8 levels in contralateral lung, and reduced IL-8, IL-10, TNFα, and SDF-1α levels in the injured lungs [16]. In severe trauma (femur fracture and hemorrhage) swine model, UB administration 40 min after bilateral femur fracture plus hemorrhage significantly reduced fluid requirements to maintain a mean arterial pressure by more than 60%. This was associated with transient immunodepression as observed by reduced endotoxin-induced TNFα production [15]. In controlled cortical impact injury in rats, UB treatment enhanced ED1+ cell (marker for macrophage/microglia) infiltration in the pericontusional cortex and altered IL-10 expression in the contusion 72 h after injury [97]. In a rat model of lung I/R injury, UB treatment enhanced the Th2 cytokine response in post-ischemic lungs during reperfusion as evidenced by increased concentrations of IL-4, IL-13, and IL-10 in lung homogenates. Here, UB treatment reduced lung edema formation and improved pulmonary function during lung I/R injury [99]. In a mouse model of myocardial I/R injury, UB treatment reduced numbers of infiltrating neutrophils and macrophages in the infarcted LV region and improved heart function 3 days post-I/R. Circulating levels of IL-12 were significantly lower in UB-treated I/R group versus I/R [17]. UB treatment was also associated with improved heart function 28 days post-I/R injury in mice. Circulating levels of IL-6, G-CSF, and IL-2 were lower in UB-treated I/R group vs. I/R [96]. In human PBMNCs, which can include monocytes that are precursors to macrophages, eUB inhibited TNF-α expression and secretion in response to LPS [14]. Interestingly, eUB had no effect on TNF-α secretion in PBMCs in response to other pro-inflammatory stimuli including zymosan A and S aureus [14]. In contrast, eUB treatment synergistically upregulated TNF-α gene transcription and secretion in the presence of LPS in RAW264.7 macrophage cell line [100]. Collectively, these studies provide evidence that UB treatment has the potential to influence inflammatory response following tissue injury. However, the mechanisms by which eUB modulates the immune response remain to be investigated.

### 7.2. Exogenous UB: Role in Macrophage Phenotype and Function

**Figure 4:**
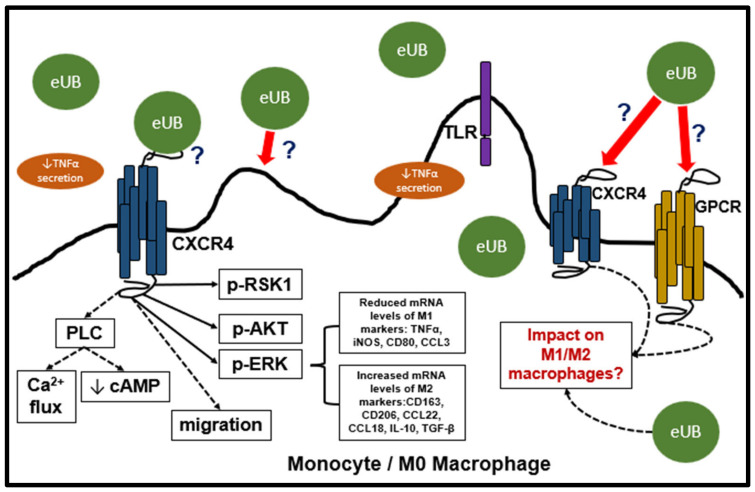
Proposed role of eUB in phenotype and function of macrophages in the heart post-MI. In THP-1 monocytes, eUB/CXCR4 signaling can potentially activate phospholipase Cβ (PLC), promote intracellular Ca^2+^ flux and reduce cAMP levels. eUB/CXCR4 axis can activate RSK1, Akt, and ERK, and influence migration. In M0 macrophages, eUB affects expression of genes associated with M1 and M2 polarization.

A few studies provide evidence that UB treatment has the potential to influence intracellular signaling, phenotype, and function of monocytes/macrophages. In the THP-1 human monocytic cell line, a precursor for macrophages, eUB has been reported to bind to and be internalized with CXCR4 [83]. In these cells, eUB/CXCR4 signaling reduced cAMP levels and promoted intracellular calcium flux, likely through the activation of phospholipase Cβ [83]. UB/CXCR4 signaling induced transient phosphorylation/activation of ERK1/2, RSK1, and Akt in THP-1 cells and promoted chemotaxis [101]. In M0 macrophages (non-activated macrophages), UB treatment resulted in a change in the phenotype. The macrophages became elongated, flat, and branching compared to control groups. In addition, UB treatment reduced TNF-α secretion [21]. UB treatment promoted hepatoma cell metastasis in the lung and increased the ratio of M2 macrophages in the lung tissue and peripheral blood of tumor-bearing mice [20]. In vitro UB treatment of M0 macrophages decreased mRNA levels of M1 polarization markers such as TNF-α, iNOS, CD80, and CCL3; while upregulating markers associated with M2 polarization such as CD163, CD206, CCL22, CCL18, IL-10, and TGF-β [20]. Within the heart, the inflammatory response with respect to the number of neutrophils and macrophages was significantly lower in the UB-treated group 3 days post-I/R [17]. Collectively, these studies provide evidence that UB/CXCR4 signaling has the potential to modulate macrophage migration, phenotype, and function (Figure 4).

## 8. Future Directions

Significant strides have been made in understanding the role of macrophages in post-MI infarct repair and healing. However, future investigations are needed to fully understand the role of macrophages in post-MI remodeling. Macrophages are considered as mediators of the acute and reparative inflammatory phase post-MI. Therefore, proper timing of their migration and polarization towards M2 phenotype may hold promise in wound repair in the post-MI setting. Macrophages are known to affect the function of other cell types of the heart to promote angiogenesis and scar formation late post-MI. A mechanistic understanding of how macrophages are capable of influencing the function of other cell types of the heart may provide opportunities to identify new therapeutic targets. Efferocytosis appears to be essential for wound clearance and improved myocardial remodeling. Could it be possible to enhance the process of efferocytosis to limit the post-MI damage to the heart? In vitro studies have identified several factors involved in polarization of monocytes towards M1 and M2. However, the in vivo post-MI tissue environment is complex, and multiple factors may come into play to determine overall macrophage phenotype. A thorough understanding of these factors may be warranted to define the role of macrophages in the remodeling processes of the heart post-MI.

A limited number of studies provide evidence for the role eUB in modulation of phenotype and function of macrophages. Further research to elucidate the role of eUB in activated macrophage phenotypes, polarization, signaling, migration, and phagocytosis may help in defining the therapeutic potential of eUB in the heart post-MI. eUB has been shown to signal through CXCR4 in monocytes, a precursor cell to macrophages, and affect M0 non-activated macrophages. In these cells, eUB activated cell signaling pathways, altered mRNA and protein levels associated with M1/M2 polarization, and affected chemotaxis [20,21,83,101]. However, there is limited knowledge about the impact of eUB on cardiac macrophages, and how this could affect the immune response post-MI. Additionally, the specific mechanism that eUb uses to signal in macrophages still needs to be elucidated. Currently, CXCR4 has been identified as a receptor for eUB. However, this does not exclude the possibility of eUB signaling through other GPCRs, as it is common that individual ligands are able to signal through multiple receptor complexes (Figure 4). Of note, CXCR4 is known to heterodimerize with other receptors, such as CXCR7, β2-AR, and α1-AR [7]. The uptake and internalization of eUB in macrophages may also affect their phenotype and function via ubiquitination of cellular proteins. Therefore, further investigation into the impact of eUB on macrophages following MI may provide vital insights into the physiological impacts of eUB signaling, and the mechanism by which eUB may modulate the inflammatory response post-MI.

## 9. Conclusions

The inflammatory phase is a regulator of repair during the remodeling process in the heart post-MI that can become exacerbated and lead to excessive cell death and further cardiac injury [2]. Macrophages are essential during the inflammatory phase, as the depletion of macrophages leads to adverse cardiac remodeling and increased rates of mortality [67]. Macrophages are vital mediators of inflammation and repair through processes such as migration to the site of injury, inflammatory signaling, phagocytosis, ECM degradation, and anti-inflammatory signaling towards proliferation, angiogenesis, and scar formation [44,45,51]. Several studies now provide evidence that eUB has immunomodulatory effects. In the plasma of patients post-MI, levels of UB are shown to be increased [13]. In a mouse model, treatment with eUB reduces inflammation and tissue injury, including reduced infiltration of macrophages and preserved cardiac function 3 days post-I/R injury of the heart [17]. Understanding of the effect of eUB macrophages in different phases of infarct healing may help towards development of new therapies to attenuate adverse cardiac remodeling post-MI.

## Data Availability

No new data was created or analyzed in this study. Data sharing is not applicable to this article.
